# JBO 2021 List of Reviewers

**DOI:** 10.1117/1.JBO.27.1.010101

**Published:** 2022-01-20

## Abstract

Thanks to reviewers who served JBO in 2021.

The *Journal of Biomedical Optics* sincerely thanks the following individuals who served as reviewers in 2021. The success of our publication hinges on the voluntary contributions of time and energy put forth by these professionals.

Marwa Abdelaziz
Osman Ahsen
Alireza Akbarzadeh
Melissa Aldrich
Alba Alfonso-Garcia
Anna Letizia Allegra Mascaro
Robert Amelard
Vijayakumar Anand
Marco Andreana
Alexander Antaris
Matthew Applegate
Hidenobu Arimoto
Xavier Attendu
Kamran Avanaki
Maxime Baillot
Wesley Baker
Timothy Baran
Luca Baratelli
Randall Barbour
Margarida Barroso
Connor Barth
Adam Bauer
Peter Baumhoff
Kathy Beaudette
Marzia Bedoni
Muyinatu Bell
Mikhail Berezin
Kirstine Berg-Sorensen
Vaughn Betz
Richard Blackmon
Giles Blaney
Walter Blondel
Sarah Bohndiek
Ekaterina Borisova
Matthew Brenner
Yang Bu
Maria Burdanova
Miran Burmen
David Busch
Sergio Calixto
Colin Campbell
Rui Cao
Xu Cao
Stefan Carp
Jeffrey Cassidy
Jonathan Celli
Andrew Chalmers
Maysam Chamanzar
Defu Chen
Mingzhou Chen
Nanguang Chen
Nikita Chernomyrdin
Raghav Chhetri
Ata Chizari
Bernard Choi
Shiwei Chu
Joseph Chue-Sang
Olga Conde
Daniel Cote
Rodrigo Cuenca
Arnaud Cuisset
Andrea Curatolo
Anabela Da Silva
Sajan Daniel George
Dhiman Das
Rupsa Datta
Elizabeth Davenport
Arie de Jong
Elbert De Josselin de Jong
Hamid Dehghani
Laura Di Sieno
Michele Diana
Charles DiMarzio
Irina Dolganova
James Dormer
Alexander Doronin
Alexandre Douplik
Meghan Driscoll
Mathieu Ducros
Nicholas Durr
Adam Eggebrecht
Andrew Ekpenyong
Daniel Elson
Olga Eremina
Thomas Ertl
Conor Evans
Qianqian Fang
Felix Fanjul-Velez
Sergio Fantini
Hamid Farrokhi
Baowei Fei
Peng Fei
Jinchao Feng
Farzad Fereidouni
Martin Fischer
Dror Fixler
Florian Foschum
Jan Erik Freund
Daniel Fried
Mitsuhiro (Hiro) Fukuda
Dragana Gabric
Fei Gao
Shan Gao
Yuanyuan Gao
Arsenii Gavdush
Michael Giacomelli
Jonas Gienger
Daniel Gil
Anuradha Godavarty
Michalina Gora
Dimitris Gorpas
Janek Gröhl
Zhengping Guan
Jacqueline Gunther
Min Guo
Rachael Hachadorian
Takafumi Hamaoka
Martin Hammer
Matthew Hancock
Lida Hariri
Andrew Harvey
Carole Hayakawa
Yoshio Hayasaki
Qinghua He
Shenghua He
Tiffany Heaster
Wido Heeman
Patrick Helfenstein
Maria del Socorro Hernandez-Montes
Brian Hill
Abraham Hirshberg
Jens Horstmann
Chenfei Hu
Jie Hui
Brady Hunt
Tiancheng Huo
Nicusor Iftimia
Vinay Ingle
Miya Ishihara
Raeef Istfan
Minbiao Ji
Mengyu Jia
Shuliang Jiao
Renu John
Jesse Jokerst
Tschackad Kamali
Dongkyun Kang
Stephen Kanick
Karol Karnowski
Kavon Karrobi
Bhuvaneshwari Karunakaran
Deepa Kasaragod
Gleb Katyba
Gopinath Kaundinya
Bjoern Kemper
Necla Kenar
Masashi Kiguchi
Ki Hean Kim
Yury Kistenev
Peter Kner
Gennady Komandin
Sergey Komarov
Kivanc Kose
Ivan Kosik
Sri-Rajasekhar Kothapalli
Nikola Krstajic
Konstantin Kudrin
Anand Kumar
Sanjeev Kumar
Puxiang Lai
Hengrong Lan
Kirill Larin
Vladimir Lasarev
Marcel Lauterbach
Ekaterina Lazareva
Lawrence Le
V. N. Du Le
Aymeric Le-Gratiet
Byeong Ha Lee
Robert Lee
Seung Ah Lee
Terence Leung
Changhui Li
Changqing Li
Jianwei Li
Jiasong Li
Lei Li
Mucong Li
Peng Li
Shuying Li
Ting Li
Zhe Li
Antonia Lichtenegger
Chihsiang Lien
Lothar Lilge
Weichiang Lin
Norman Lippok
Maritoni Litorja
Gangjun Liu
Hanli Liu
Xin Liu
Yang Liu
David Orlando Grajales Lopera
Jose Miguel Lopez-Higuera
Pablo Loza-Alvarez
Guolan Lu
Rongwen Lu
Geoffrey Luke
Cheng Ma
Hui Ma
Calum MacAulay
Boris Majaron
Shuichi Makita
Subhamoy Mandal
Michael Mandella
Zbignevs Marcinkevics
Oszust Mariusz
Geminiano Martinez-Ponce
Amaan Mazhar
David McGloin
Robert McLaughlin
Igor Meglinski
Guanghan Meng
Elisa Michelini
Vicente Mico
Mohammad Moeini
Bruno Montcel
Stephen Morgan
Luke Mortensen
Moein Mozaffarzadeh
Timothy Muldoon
Peter Munro
Sangeeta Murugkar
Oleg Nadyarnykh
Sreyankar Nandy
John Quan Nguyen
Van Phuc Nguyen
Fanny Nina-Paravecino
Bo Ning
David Nolte
Takanori Nomura
Ioan Notingher
Jeffry Nyman
Marien Ochoa Mendoza
Shinpei Okawa
Luis Oliveira
Ilya Ozheredov
Gregory Palmer
Guenther Paltauf
Maria Papadaki
Arturo Pardo
Evgueni Parilov
Il-Yong Park
YongKeun Park
Souradip Paul
Yannis Paulus
Vijitha Periyasamy
Artem Perov
Elena Petrova
T. Joshua Pfefer
Thao Pham
Daqing Piao
Mark Pierce
Antonio Pifferi
Michael Pircher
Arthur Plante
Nancy Pleshko
Raju Poddar
Martin Poenie
Brian Pogue
Mary Potasek
Eric Potma
Chetan Poudel
Jaya Prakash
Guillem Pratx
G. L. Puyo
Le Qiu
Xiangyu Quan
Matin Raayai Ardakani
Narasimhan Rajaram
Rajendran Rajeev
Suhrud M Rajguru
Julio Ramirez-San Juan
Janaka Ranasinghesagara
Lise Randeberg
Aleksandra Rapacka-Zdonczyk
Karen Reiser
Darren Roblyer
Jeremy Rogers
Kelly Rogers
Sandra Rugonyi
Alberto Ruiz
Rolf Saager
Guennadi Saiko
Inga Saknite
Danuta Sampson
David Sampson
Angelo Sassaroli
Felix Scholkmann
Kevin Schomacker
Richard Schwarz
Sanathana Konugolu Venkata Sekar
Sergey Seliverstov
Janaka Senarathna
Eva Sevick-Muraca
Babak Shadgan
Ruibo Shang
Yu Shang
Arunima Sharma
Douglas Shepherd
Benjamin Sherlock
Manmohan Singh
Dmitry Sitnikov
Koneswaran Sivagurunathan
Kathyayini Sivasubramanian
Jason Smith
Zachary Smith
Laura Sordillo
Jonathan Sorger
Janis Spigulis
Ronald Sroka
Herbert Stepp
Samuel Streeter
Tomas Stromberg
Narayanan Subhash
Ulas Sunar
John Sunwoo
Syeda Tabassum
Jack Tang
Yubo Tang
Xiaodong Tao
Yuankai Tao
Tanja Tarvainen
Dieter Richard Taubert
Jeffrey Thatcher
Chao Tian
Jie Tian
Lei Tian
Kenneth Tichauer
Karissa Tilbury
Jerilyn Timlin
Vassiliy Tsytsarev
Raphael Turcotte
Sergey Ulyanov
Nestor Uribe-Patarroyo
Pablo Valdes
Nynke van den Berg
Ton Van Leeuwen
Labrinus van Manen
Gijs Van Soest
Jenu Varghese Chacko
Demetrios Vavvas
Alberto Vazquez
Rudolf Verdaasdonk
William Vogt
Heidrun Wabnitz
Mark Wainwright
Vincent Wallace
Hao Wang
Hongda Wang
Lin Wang
Liqiang Wang
Quan Wang
Shang Wang
Dale Waterhouse
Jessie Weber
Robert Weersink
Wei Wei
Gentiana Wenzel
Brian Wilson
Jesse Wilson
Binlin Wu
Yicong Wu
Lei Xi
Wenfeng Xia
Qiwei Xie
Guan Xu
Vladislav Yakovlev
Phaneendra Yalavarthy
Seiichi Yamamoto
Bin Yang
Eric Yang
Junjie Yao
Xincheng Yao
Yusuf Yaras
Arjun Yodh
Jie Yuan
Yaoshen Yuan
Vladimir Zaitsev
Robert Zawadzki
Haishan Zeng
Menghao Zhang
Miao Zhang
Pengfei Zhang
Wei Zhang
Yide Zhang
Hubin Zhao
Yanyu Zhao
Guoan Zheng
Wei Zheng
Chao Zhou
Qifa Zhou
Jiang Zhu
Shouping Zhu
Yizheng Zhu

